# Blunt cerebrovascular injury in elderly fall patients: are we screening enough?

**DOI:** 10.1186/s13017-018-0188-z

**Published:** 2018-07-04

**Authors:** Vincent P. Anto, Joshua B. Brown, Andrew B. Peitzman, Brian S. Zuckerbraun, Matthew D. Neal, Gregory Watson, Raquel Forsythe, Timothy R. Billiar, Jason L. Sperry

**Affiliations:** Division of General Surgery and Trauma, Department of Surgery, University of Pittsburgh Medical Center, 200 Lothrop Street, Pittsburgh, PA 15213 USA

**Keywords:** Blunt cerebrovascular injury, Elderly, Falls, Screening, Incidence, Intravenous contrast

## Abstract

**Background:**

Blunt cerebrovascular injuries (BCVI) are generally associated with high-energy injury mechanisms. Less is known regarding lower-energy injuries in elderly patients. We sought to determine the incidence of BCVI and characterize current BCVI screening practices and associated complications in elderly ground-level fall patients (EGLF, ≥ 65 years). We hypothesized that BCVI in EGLF patients would be clinically significant and screening would be less common.

**Methods:**

A retrospective study was performed utilizing the National Trauma Data Bank (NTDB, 2007–2014) and single institutional data. BCVI risk factors and diagnosis were determined by ICD-9 codes. Presenting patient characteristics and clinical course were obtained by chart review. The NTDB dataset was used to determine the incidence of BCVI, risk factors for BCVI, and outcomes in the EGLF cohort. Local chart review focused on screening rates and complications.

**Results:**

The incidence of BCVI in EGLF patients was 0.15% overall and 0.86% in those with at least one BCVI risk factor in the NTDB. Upper cervical spine fractures were the most common risk factor for BCVI in EGLF patients. In EGLF patients, the diagnosis of BCVI was an independent risk factor for mortality (OR1.8, 95% C.I. 1.5–2.1). The local institutional data (2007–2014) had a BCVI incidence of 0.37% (*n* = 6487) and 1.47% in those with at least one risk factor (*n* = 1429). EGLF patients with a risk factor for BCVI had a very low rate of screening (44%). Only 8% of EGLF patients not screened had documented contraindications. The incidence of renal injury was 9% irrespective of BCVI screening.

**Conclusions:**

The incidence of BCVI is clinically significant in EGLF patients and an independent predictor of mortality. Screening is less common in EGLF patients despite few contraindications. This data suggests that using age and injury mechanism to omit BCVI screening in EGLF patients may exclude an at-risk population.

**Trial registration:**

IRB approval number: PRO15020269. Retrospective trial not registered

## Background

Blunt cerebrovascular injury (BCVI) is an injury to the carotid or vertebral arteries which can result in devastating consequences. BCVI is estimated to occur in 1–2% of blunt traumatic hospital admissions [1–3]. Appropriate screening is of paramount importance due to the morbidity and mortality of ischemic events attributable to BCVI if not diagnosed and properly managed [1, 4]. Detection of BCVI before the onset of symptoms allows for appropriate treatment and greatly reduces the risk of neurological sequelae [5]. There have been considerable research efforts made to determine the appropriate risk factors for BCVI that warrant screening. Current screening guidelines for BCVI are based in part on anatomic risk factors such as cervical spine injuries and basilar skull fractures [6–8].

Despite the general research interest in BCVI, the injury has not been specifically investigated in elderly patients. Elderly trauma admissions have increased as the population of Americans over the age of 65 continues to grow [9]. Injuries in the elderly often involve low-energy mechanisms [10]. Fall injuries are particularly common in the elderly population [9]. Low-energy injuries in the elderly often involve risk factors for BCVI that would generally mandate screening. In clinical practice, BCVI screening is often associated with higher-energy injury mechanisms which may predispose practitioners not to screen as commonly in low-energy injuries [8]. The risk of BCVI in these low-energy injuries has not been adequately characterized. Due to limited knowledge regarding the incidence of BCVI in elderly trauma patients, screening may be less prevalent in the elderly.

We undertook a retrospective review of the National Trauma Data Bank (NTDB 2007–2014) as well as the local institutional registry data at the University of Pittsburgh (2007–2014) to characterize BCVI in elderly trauma patients, particularly those with low-energy fall injuries. The study aims to define the incidence of BCVI in this patient cohort and explore the frequency of risk factors for BCVI. Screening rates, complications of screening for BCVI in the elderly, and outcomes are also investigated to see if current practices need to be modified to provide better care to this subset of patients. We hypothesized that the incidence of BCVI in low-energy falls would be clinically significant in the elderly population and screening rates would be lower relative to younger patients and elderly patients with non-fall injury mechanisms.

## Methods

We conducted a retrospective review of two large datasets from 2007 to 2014 using only blunt injured patients. Patients were divided by age with elderly patients being considered to be at least 65 years of age at the time of admission. The National Trauma Data Bank (NTDB) is a collection of data from over 900 US trauma centers [11]. Over 1 million elderly patients with blunt injuries were used to determine the incidence of BCVI in the elderly population, specifically those involved in low-energy mechanisms of injury. Prominent risk factors that could be obtained from the data set were analyzed to examine their relationship to the incidence of BCVI.

The incidence of BCVI in all blunt patients was obtained from the NTDB using ICD-9 codes for BCVI injuries (900.00, 900.01, 900.03, 900.82, 900.89, 900.90). Elderly ground-level fall (EGLF) patients were defined by those with low-energy falls using ICD-9 E-codes specific for such injury mechanisms (880.1, 884.2–884.6, 885.9, 888.1, 888.8, 888.9). Falls from a height and falls down stairs were not included in the EGLF group as they were considered high-energy injury mechanisms. Such high-energy falls and any other non-fall blunt trauma were defined as the elderly non-GLF group. Risk factors for BCVI that could be extracted from the NTDB included cervical spine injuries (fractures and subluxations), basilar skull fractures, Le Fort II and III fractures, and mandible fracture [7]. These were selected using corresponding ICD-9 codes (801.0–801.9, 802.2–802.39, 805.0–805.18, 806.0–806.19, 839–839.18). Upper cervical spine fractures were defined as fractures in vertebrae 1–3 and lower spine were fractures in cervical vertebrae 4–7. This was done to reflect the difference in risk for BCVI depending on the location of the cervical spine fracture [7].

The incidence of BCVI was determined in young patients 18–64 years of age with or without a GLF mechanism, elderly non-GLF patients, and EGLF patients for all blunt injury patients and in those patients with at least one screening risk factor for BCVI. We then compared EGLF patients who suffered BCVI to those EGLF patients without BCVI to characterize differences between the two groups. Finally, we utilized logistic regression to determine if BCVI in EGLF patients was independently associated with mortality in this cohort after controlling for confounding factors.

Local data from the University of Pittsburgh Medical Center trauma registry, an urban level 1 trauma center with 5000 trauma patients per year, was used to determine incidence and characteristics of patients who suffered BCVI, associated outcomes, screening rates, and complications associated with screening. Our institution had a BCVI screening protocol during the time of the study (2007–2014) which followed the most up-to-date published guidelines [7].

BCVI incidence from the local institutional data was determined by selecting all patients ≥ 18 years of age with an ICD-9 code for BCVI. The incidence of BCVI in younger patients, elderly non-GLF patients, and EGLF patients with and without risk factors for BCVI was determined. Specific screening practices, complications from screening, and outcomes were then obtained via chart review. BCVI injury grade was defined by the BCVI grading scale [12]. Radiology reports that indicated that the injury was more likely due to a pre-existing process (atherosclerotic changes) were not included in the positive BCVI groups.

Patients with specific risk factors for BCVI were examined to determine screening rates at our institution. Upper cervical spine fractures were studied in depth as this injury complex was the most common risk factor for BCVI in the elderly. Additionally, local institutional screening protocol recommends screening for all upper cervical spine fractures. Screening rates were calculated by selecting all patients with an upper cervical spine fracture who survived for at least 24 h. Rates of BCVI screening with computed tomography angiography (CTA), magnetic resonance angiography (MRA), or digital subtraction angiography (DSA) within 72 h of admission were determined via chart review. Time until BCVI screening was defined as the amount of time between admission and BCVI radiologic screening exam. Patients with BCVI screening were compared to those that were not screened for all patients with upper cervical spine fractures. Demographics, injury characteristics, renal function (estimated glomerular filtration rate [eGFR]), and pre-injury anti-thrombotic (aspirin, p2y_12_ inhibitors, heparin, warfarin, and novel oral anticoagulants) were compared between the two groups.

Lastly, we compared creatinine levels between patients screened for BCVI (*n* = 442) and a randomly selected elderly group (*n* = 200) that received no IV contrast during their hospital stay. Renal injury was defined as an increase in baseline creatinine by 25% or by 0.5 within 72 h of receiving contrast or an increase within 72 h from admission for those patients who did not receive intravenous contrast. The definition for renal injury is based upon previous studies of contrast-induced nephropathy [13, 14].

All data are presented as a mean (standard deviation [SD]), median (interquartile range [IQR]), or percentage. Univariate comparisons were made using Student’s *t* test for normally distributed data, Mann-Whitney *U* test for non-parametric data, and chi-square test for proportions. An *α* of 0.05 was considered significant. Multivariate comparisons for mortality were performed by logistic regression and adjusted for baseline demographics, injury severity, pertinent associated injuries, and presenting vital signs. The *C* statistic was used to characterize model discrimination and calibration curves were used to characterize model fit.

## Results

There were over 1.2 million blunt trauma patients aged 65 and older in the NTDB dataset during the time period of this study. BCVI injuries and associated injury mechanisms were selected using appropriate ICD-injury codes and E-codes respectively. Ground-level falls accounted for 67% of blunt traumatic injuries in the ≥ 65-year-old cohort. EGLF injuries were found to have an overall BCVI incidence of 0.15% in the elderly cohort (Table 1). This was significantly lower than the incidence in younger patients. Despite the overall lower incidence, EGLF injuries accounted for 33% of the cases of BCVI in the elderly cohort due to the prevalence of this injury mechanism. Less than 5% of BCVI injuries in the younger population were a result of low-energy falls.

**Table 1:** Incidence of BCVI based upon ICD-9 code from the NTDB (2007–2014), stratified by age (18–64, 65+) and injury mechanism

	All blunt injuries	≥ 1 risk factor for BCVI	*p*
18–64 non-ground-level fall	0.70% (14497)	2.8% (10758)	< 0.001
18–64 ground-level fall	0.20% (715)	1.1% (388)	< 0.001
Elderly non-ground level fall	0.59% (2330)*	2.49% (1810)*	< 0.001
Elderly ground level fall	0.15% (1168)† ‡	0.86% (652)† ‡	< 0.001

All data are presented as incidence (number of patients with BCVI)

*Statistically significant difference relative to 18–64 non-ground-level fall group (*p* < 0.05)

†Statistically significant difference relative to elderly non-ground-level fall group (*p* < 0.05)

‡Statistically significant difference relative to 18–64 ground-level fall group (*p* < 0.05)

When selecting patients with at least one risk factor for BCVI using specific ICD-9 injury codes, the incidence of BCVI increased in all groups as expected. The incidence in patients with EGLF injuries was almost six times higher with at least one injury risk factor being present (Table 1).

EGLF patients with and without documented BCVI injuries were compared (Table 2). Patients with BCVI were more commonly male, had higher injury severity, and more commonly had injuries associated with BCVI. Upper cervical spine fractures were common in EGLF BCVI patients, occurring in 32% of patients. This is much higher than any other risk factor screened for in the NTDB. Cervical spine injuries in general (fractures or subluxations anywhere in the cervical spine) occurred in 45% of BCVI patients. While risk factors for BCVI are much more prevalent in patients with BCVI, they still are frequently present in EGLF patients who did not have BCVI injury codes. Mortality was significantly higher in patients with BCVI.

**Table 2:** Elderly ground-level fall (EGLF) patient comparison with and without documented BCVI

	BCVI injury		
	No	Yes	
	*n* = 796,021	*n* = 1168	*p*
Age (years)	81.0 (7.5)	80.2 (7.4)	< 0.001
Male sex	33.0%	45.8%	< 0.001
Admission GCS	15 (15-15)	15 (14-15)	< 0.001
Admission SBP (mmHg)	149 (29)	151 (33)	0.007
Upper cervical spine fracture	3.7%	31.9%	< 0.001
Lower cervical spine fracture	1.3%	9.0%	< 0.001
Any cervical spine injury	5.6%	44.5%	< 0.001
Basilar skull fracture	2.5%	11.2%	< 0.001
Le Fort fracture	1.9%	5.50%	< 0.001
Mandible fracture	0.4%	0.60%	0.371
At least 1 injury risk factor for BCVI	9.1%	56.0%	< 0.001
Greater than 1 risk factor for BCVI	1.2%	8.30%	< 0.001
Mortality	5.0%	19.0%	< 0.001

Data are presented as mean (SD), percentage, or median (IQR). *p* values are calculated by Mann Whitney *U* test or chi-square test

Multivariate logistic regression was used to determine if BCVI was an independent risk factor for mortality in the EGLF cohort after controlling for important confounders (Table 3). BCVI was significantly associated with over 77% higher odds of mortality after adjusting for demographics, injury severity, and other injuries which are risk factors for BCVI screening. Visual inspection of the model calibration plot demonstrated excellent calibration as the observed and predicted mortality correlated closely across predicted mortality risk deciles.

**Table 3:** Logistic regression model to determine independent risk factors of in-hospital mortality in elderly ground-level falls (*n* = 1168)

	Coefficient	S.E.	Wald	Odds ratio	95% C.I.	*p*
Age (years)	0.046	.001	2525	1.047	1.045–1.049	< 0.001
Male sex	0.483	.013	1442	1.621	1.581–1.662	< 0.001
ISS	0.091	.001	12,574	1.096	1.094–1.097	< 0.001
Admission SBP (mmHg)	− 0.005	.000	720	0.995	0.994–0.995	< 0.001
Admission GCS	− 0.280	.002	25,836	0.756	0.753–0.758	< 0.001
BCVI	0.571	.097	35	1.770	1.464–2.139	< 0.001
≥ 1 BCVI screening injury risk factor	0.379	.017	495	1.461	1.413–1.511	< 0.001
Constant	− 3.401	.082	1720			< 0.001

Logistic regression model for predictors of in-hospital mortality. *p* values are calculated by the Wald test. Area under the cross-validated receiver operating characteristic curve for the model is 0.8233

*CI* confidence interval, *S*E standard error

In the 6520 EGLF patients at our institution, the incidence of BCVI was 0.37%; this was significantly lower compared to that of younger patients and elderly patients with high-energy injury mechanisms (Table 4). There was no significant difference in BCVI incidence when comparing the 18–64 age group to the elderly non-GLF group. The trends of BCVI incidence based upon injury mechanism and risk factors were similar when comparing the local institutional data to the NTDB data. The incidence was roughly twice as high in the local data compared to the BCVI data in all groups which may signify differences between the respective data sets. When selecting only patients with risk factors for BCVI, the incidence increased significantly in all groups. The incidence remained lower in the EGLF group compared to that of the other two patient groups (Table 4).

**Table 4:** Local institution BCVI incidence data 2007–2014

	All blunt injuries	≥ 1 risk factor for BCVI	*p*
18–64	1.17% (290)	5.68% (270)	< 0.001
Elderly non-ground-level fall	1.12% (53)	4.87% (52)	< 0.001
Elderly ground-level fall	0.37% (24)*	1.47% (21)*	< 0.001

All data are presented as incidence (number of patients with BCVI). Incidence of BCVI based upon ICD-9 code from registry data from 2007 to 2014, stratified by age (18–64, 65+) and injury mechanism

*Statistically significant difference relative to elderly non-GLF (*p* < 0.05)

We characterized all elderly BCVI patients at our institution during the time period of the study (Table 5). EGLF injuries accounted for 31% of the BCVI in the institutional data. This was similar to findings in the NTDB data (33%). Upper cervical spine fractures were the most common risk factor for BCVI. Most EGLF patients had a least one risk factor for BCVI screening. The severity of BCVI included grade 1 thru grade 4 injuries. EGLF mechanism still resulted in serious BCVI injuries with 25% of the low-energy injuries resulting in vessel occluding grade 4 injuries. The incidence of cerebral ischemic events in the EGLF patients was similar to patients aged 18–64 (4.2% compared to 5.3% *p* = 0.81).

**Table 5:** Elderly ground-level fall patients with BCVI

	(*n* = 24)
Demographics	
Age (years)	81.6 (7.6)
Male sex	50%
ISS	9.5(5.8–13)
Admission GCS	15 (14–15)
Pre-injury anti-thrombotic	75.0%
Upper cervical spine fracture	79.2%
Any risk factor for BCVI	87.5%
BCVI location	
Carotid	25%
Vertebral	75%
Grade	
1 (intimal irregularity with < 25% narrowing)	50%
2 (dissection, intramural hematoma, or intimal flap with > 25% narrowing)	12.5%
3 (pseudoaneurysm)	12.5%
4 (vessel occlusion)	25%
Treatment	
Aspirin	16
Aspirin + clopidogrel	1
Heparin	2
Stenting + aspirin	1
None	4
Outcome	
BCVI attributable stroke	4.2%
Mortality	8.3%

Data presented as a mean (SD), median (IQR), or percentage of the patient population

**Fig. 1:**
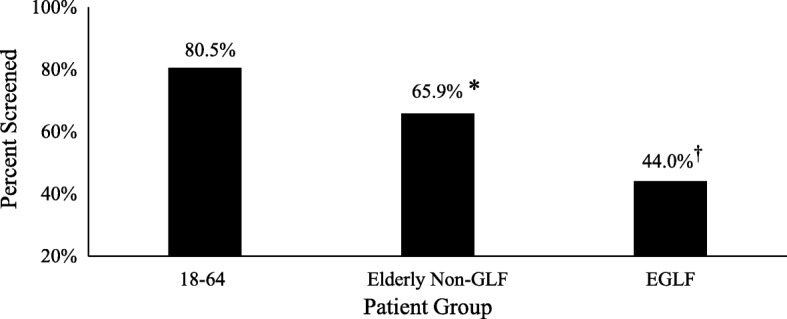
Screening rates for BCVI with known upper cervical spine fracture. *p < 0.05 relative to 18–64-year-old group; ^†^*p* < 0.05 relative to elderly non-GLF group

The NTDB and local institutional data indicated that patients with cervical spine fractures were at a high risk for BCVI. All patients with ICD-9 codes (805.01–805.03) for upper cervical spine fractures were then selected from the local trauma registry. Appropriate BCVI screening (CTA, MRA, DSA) for these 1387 patients was then determined via chart review. BCVI screening rates differ significantly based on age and injury mechanism. In elderly non-GLF patients, screening was 65.9% with a decrease to 44.0% in EGLF patients (Fig. 1).

Elderly patients with upper cervical spine fractures who were screened for BCVI were compared to those who were not screened (Table 6). Patients not screened for BCVI were significantly older and more likely to have suffered a ground-level fall. Patients who did not undergo BCVI screening were more likely to have compromised renal function on initial laboratory assessment. An eGFR of < 30 mL/min/1.73 m^2^ was used as a surrogate for renal insufficiency and is considered a relative contraindication for IV contrast at our institution. Rates of pre-injury anti-thrombotic medication were not different between those screened for BCVI and those not screened.

**Table 6:** Comparison of elderly patients with upper cervical spine fractures with and without BCVI screening

	Screened		
	Yes	No	
	*n* = 442	*n* = 412	*p*
Age (years)	79.9 (8.0)	83.9 (8.2)	< 0.001
Male sex	39.0%	41.0%	0.44
ISS	9 (5–14)	9 (5–13)	0.32
Admission GCS	15 (15–15)	15 (14–15)	< 0.001
Admission SBP (mmHg)	151 (30)	149 (29)	0.67
Pre-injury anti-thrombotic	66.0%	64.0%	0.41
EGLF injury	54.7%	72.0%	< 0.001
Admission eGFR < 30 (mL/min/1.73 m^2^)	2.70%	8.00%	< 0.001
Time to BCVI screening (hours)	9 (14.3)	N/a	N/a
Mortality	7.0%	12.6%	0.006

Data are presented as mean (SD), median (IQR), or percentage of the patient population. *p* values are calculated by Mann Whitney *U* test or chi-square test

Patients who received IV contrast (*n* = 442) for BCVI screening were compared to a randomly selected group of elderly trauma patients who did not receive IV contrast (*n* = 200). Rates of renal injury did not differ between those who received IV contrast for BCVI screening compared to those patients who did not receive contrast from the random sample (8.7 vs 9.4% *p* = 0.84). The injury severity score was higher in the group that underwent BCVI screening (median 10 vs 9, *p* = 0.04). Of the 8.7% (*n* = 38) of patients who were screened and had renal injury, seven patients had persistent increases in their creatinine for over 1 week and a single patient required initiation of dialysis.

## Discussion

The diagnosis and incidence of BCVI have increased over time corresponding with documented increased screening rates [15, 16]. Despite this increased screening and the growing elderly population in the USA, little is known about risk of BCVI in elderly patients with low-energy injury mechanisms, particularly ground-level falls [8]. Such low-energy injuries are associated with higher rates of fractures and other complications compared to younger patients [10, 17, 18]. This is due to osteopenia and altered biomechanics in elderly patients [18, 19]. We speculated that these low-energy mechanisms would still result in injuries such as cervical spine and basilar skull fractures which would place patients at risk for BCVI.

The current analysis provides evidence for the decreased incidence of BCVI in elderly ground-level fall patients compared to patients with other injury mechanisms. However, those patients with risk factors for BCVI screening had an incidence of BCVI similar to the general trauma population approaching 1–2% [1–3]. Approximately 1 in 10 EGLF patients had at least one risk factor for BCVI in the current study. The diagnosis of BVCI was found to be an independent risk factor for mortality in the EGLF cohort after controlling for other important variables which impact mortality. BCVI was associated with a 4.2% rate of cerebral ischemic events in the EGLF patients.

Despite having a well-defined BCVI screening protocol at our institution, screening rates were significantly lower in EGLF patients even when injuries known to be BCVI risk factors were present. Screening was significantly less common in elderly patients compared to that in younger patients regardless of injury mechanism. Providers may justify omitting BCVI screening for many reasons: if patients are already on BCVI treatment (anti-thrombotic therapy), fear of contrast-induced nephropathy, and lack of perceived benefit due to assumptions that elderly patients are at low risk for having BCVI. The current study suggests that many of the above reasons to omit screening elderly patients may not be evidence-based.

When examining BCVI screening, there was no difference in the proportion of patients already on pre-injury anti-thrombotic medication. Contrast-induced nephropathy does not appear to be a reason to avoid screening if risk factors for BCVI are present. There was no significant difference in renal injury between patients who received IV contrast for BCVI screening relative to those without IV contrast imaging. This is consistent with previous studies that demonstrate that the risk of contrast-induced renal injury is low and that rates of renal injury are not different from patients who do not receive contrast [13, 14]. The patients in the unscreened group were found to have significantly higher mortality. This is likely attributable to older age and increased comorbidities in this patient group. Lastly, this study indicates that elderly patients have a lower incidence of BCVI but are still at clinically significant risk. Even with a low-energy injury mechanism, EGLF patients have a BCVI incidence of 0.86–1.47% when a screening risk factor was present.

As seen in the local data, patients can have high-grade BCVI injuries and ischemic events regardless of injury mechanism. All cases of cerebral ischemia occurred in patients before proper treatment was initiated. This is consistent with other published data on BCVI [4, 5]. Early screening in patients with risk factors can allow for proper treatment and prevention of debilitating or deadly ischemic events.

This analysis has several limitations which should be considered when interpreting the results. One major limitation of this study is the retrospective nature of the analysis. It is impossible to know how many patients had BCVI injuries but were not screened or were not properly coded for a BCVI diagnosis. It is known that BCVI incidence has increased over time with increased screening [15, 16]. We can speculate that the true incidence of BCVI in the elderly is significantly higher than what was determined in this study and future estimates will need to account for low screening rates seen in the elderly population. Lower screening rates likely contribute to the lower incidence in EGLF patients compared to that in younger patients with GLF injuries.

There are inherent limitations of large national datasets like the NTDB. The NTDB includes a disproportionate number of large hospitals with younger and more severely injured patients which may skew the true incidence of BCVI in the elderly. Hospital variability in screening rates and data reporting could significantly impact the outcomes of this study. From the NTDB data, screening rates in EGLF patients could not be determined. Lower screening rates may contribute to the difference in BCVI incidence in the NTDB compared to local institutional data. Future multi-center trials would be needed to examine screening practices at other institutions. Due to the large sample size of the NTDB cohort, our group comparisons were highly statistically significant, even though some differences were not clinically different. We attempted to highlight those differences which were clinically relevant in our interpretation of the results. For our regression model, the covariates of interest were also highly significant due to the large sample size which limits the conclusions which can be formulated from the results.

We utilized our local trauma registry to overcome limitations attributable to large national datasets. This limits the applicability to other centers across the country. To compare screening rates, we focused on the most common and robust BCVI risk factor, upper cervical spine fracture, to select our cohort. This may bias our conclusions relative to other types of injuries associated with BCVI. Despite this limitation, we fully characterized the radiographic workup for over 1300 trauma patients and performed a thorough medical record review for over 850 elderly trauma patients. Radiographic diagnosis and grade of BCVI were complicated by presence of atherosclerotic disease in elderly patients. Local radiologists generally indicated which arterial abnormalities were more likely related to a pre-existing process. When comparing the effects of IV contrast on renal injury, some patients screened for BCVI had multiple scans with IV contrast which may affect the results.

## Conclusions

The novel results of this retrospective study demonstrate that despite having lower energy injuries, elderly patients remain at risk for BCVI. Current BCVI screening of elderly ground-level fall patients is less common relative to younger patients and elderly patients with high-energy injury mechanisms. This lower screening rate exists in spite of similar risks of stroke with a BCVI diagnosis. The current data demonstrates that patients who present with risk factors for BCVI, even in those who suffer low-energy injury mechanisms, are at significant risk for BCVI. Patients should be screened for the injury when feasible and safe to allow for appropriate diagnosis and treatment. Screening decisions should not be biased by age or injury mechanism when patients meet criteria for BCVI screening.

## Abbreviations

BCVI: Blunt cerebrovascular injury
CTA: Computed tomography angiography
DSA: Digital subtraction angiography
eGFR: Estimated glomerular filtration rate
EGLF: Elderly ground-level fall (age ≥ 65 years)
GCS: Glasgow Coma Scale
GLF: Ground-level fall
ICD: International Classification of Diseases
IQR: Interquartile range
ISS: Injury severity score
MRA: Magnetic resonance angiography
NTDB: National Trauma Data Bank
SBP: Systolic blood pressure
SD: Standard deviation
